# Evaluating the Ribosomal Internal Transcribed Spacer (ITS) as a Candidate Dinoflagellate Barcode Marker

**DOI:** 10.1371/journal.pone.0042780

**Published:** 2012-08-16

**Authors:** Rowena F. Stern, Robert A. Andersen, Ian Jameson, Frithjof C. Küpper, Mary-Alice Coffroth, Daniel Vaulot, Florence Le Gall, Benoît Véron, Jerry J. Brand, Hayley Skelton, Fumai Kasai, Emily L. Lilly, Patrick J. Keeling

**Affiliations:** 1 The Laboratory, Sir Alister Hardy Foundation for Ocean Science, Plymouth, United Kingdom; 2 Provasoli-Guillard National Center for Culture of Marine Phytoplankton, West Boothbay Harbor, Maine, United States of America; 3 Australian National Algae Culture Collection, The Commonwealth Scientific and Industrial Research Organisation Marine and Atmospheric Research, Hobart, Tasmania, Australia; 4 Culture Collection of Algae and Protozoa, Scottish Association for Marine Science, Oban, United Kingdom; 5 Department of Geology, State University of New York at Buffalo, Buffalo, New York, United States of America; 6 Roscoff Culture Collection, Station Biologique Roscoff, Roscoff, France; 7 Algobank-Caen, Université de Caen Basse-Normandie, Caen, France; 8 Section of MCD-Biology, School of Biological Sciences, University of Texas at Austin, Austin, Texas, United States of America; 9 Department of Marine Sciences, University of Connecticut, Groton, Connecticut, United States of America; 10 Microbial Culture Collection, National Institute for Environmental Studies, Tsukuba, Japan; 11 Biology Department, Virginia Military Institute, Lexington, Virginia, United States of America; 12 Botany Department, University of British Columbia, Vancouver, Canada; Barnard College, Columbia University, United States of America

## Abstract

**Background:**

DNA barcoding offers an efficient way to determine species identification and to measure biodiversity. For dinoflagellates, an ancient alveolate group of about 2000 described extant species, DNA barcoding studies have revealed large amounts of unrecognized species diversity, most of which is not represented in culture collections. To date, two mitochondrial gene markers, Cytochrome Oxidase I (COI) and Cytochrome b oxidase (COB), have been used to assess DNA barcoding in dinoflagellates, and both failed to amplify all taxa and suffered from low resolution. Nevertheless, both genes yielded many examples of morphospecies showing cryptic speciation and morphologically distinct named species being genetically similar, highlighting the need for a common marker. For example, a large number of cultured *Symbiodinium* strains have neither taxonomic identification, nor a common measure of diversity that can be used to compare this genus to other dinoflagellates.

**Methodology/Principal Findings:**

The purpose of this study was to evaluate the Internal Transcribed Spacer units 1 and 2 (ITS) of the rDNA operon, as a high resolution marker for distinguishing species dinoflagellates in culture. In our study, from 78 different species, the ITS barcode clearly differentiated species from genera and could identify 96% of strains to a known species or sub-genus grouping. 8.3% showed evidence of being cryptic species. A quarter of strains identified had no previous species identification. The greatest levels of hidden biodiversity came from *Scrippsiella* and the Pfiesteriaceae family, whilst *Heterocapsa* strains showed a high level of mismatch to their given species name.

**Conclusions/Significance:**

The ITS marker was successful in confirming species, revealing hidden diversity in culture collections. This marker, however, may have limited use for environmental barcoding due to paralogues, the potential for unidentifiable chimaeras and priming across taxa. In these cases ITS would serve well in combination with other markers or for specific taxon studies.

## Introduction

Dinoflagellates are an ancient and ecologically important group of algae distantly related to ciliates and apicomplexan parasites, all part of the alveolate group [1], [2]. Approximately 2000 species have been formally identified and described [3], but species identification by traditional morphological criteria in several genera is challenging and many species remain unidentified. Moreover, molecular phylogeny has shown that many morphology-based genera are paraphyletic, such as *Amphidinum* and *Gymnodinium* [4]. Other genera have been shown to be enormously diverse, for example *Symbiodinium* [5], so named because of its symbiotic relationship with corals and other invertebrates. *Symbiodinium* was once considered to represent a single species based on morphology [6], but now contains hundreds of distinct taxonomic units, most of which have not been named (for review and comprehensive phylogeny see [7]–[10]).

The sheer variety of forms and evolutionary diversity of dinoflagellates have made classification difficult and it is clear that there is a need for a standard DNA-based identification system to keep pace with the rate of discovery. The technique of DNA barcoding, where a short, standardized stretch of DNA sequence is used to identify a species, has been applied to dinoflagellates using two mitochondrial markers, the Cytochrome Oxidase I (COI) [11] and the Cytochrome Oxidase B gene (*COB*) [12], both with variable success. The range of successful species identification with these two markers was broadly similar. However, neither marker could be amplified from all dinoflagellate strains nor could they resolve common ambiguous genera to species level. In addition to *Symbiodinium*, another problematic example is the genus *Alexandrium*, a potentially toxic dinoflagellate that may form Harmful Algal Blooms (HABs) [3], [13]–[15]. Though COI solved many problems, it also failed to resolve a number of issues, in particular surrounding some of the larger and more complex genera like *Alexandrium*, where virtually no sequence variation was found. *COB* performed similarly or better in certain genera but lacks in strain database size [12]. One of the key justifications of DNA barcoding is to enable the rapid identification of HAB species and to distinguish the toxic from non-toxic strains, in addition to maintaining an accurate catalogue of cultured strains.

In this study, we set out to test a third common barcode marker, the internal transcribed spacer (ITS) units 1 and 2, which separate the small and large subunit ribosomal RNA genes, as a barcode marker using a wide variety of dinoflagellate species from ten private and public culture collections. This marker is attractive because it has been used in previous barcoding studies of eukaryotic micro-organisms with success [16]–[19], including an assessment of dinoflagellates [20], so it is relatively well represented in public databases. Moreover, it has been shown that the presence of evolutionary conserved compensatory base pair changes in ITS2 can be used to predict species accurately in metazoans [21] and some dinoflagellates [22] including *Symbiodinium* clade types [10], [23]. However, ITS is also a difficult marker technically because it is present in multiple distinct copies, with the possibility that high intra and intergenomic variation and the presence of indels that can make direct sequencing challenging and alignment difficult. Indeed, in one deep-branching dinoflagellate lineage, the Syndiniales, ITS sequences belonging to two different strains of *Hematodinium* sp. were too divergent to be aligned [24], whilst multiple paralogues were shown to be a major issue in identifying new species of *Symbiodinium*, especially in cloned sequences [25]–[27]. One prime objective of a DNA barcode marker is universal applicability. To test the utility of ITS in dinoflagellates, we assessed nearly 400 strains belonging to 78 known species. Culture collections were used as a curated source of strains that have been independently identified by taxonomists and because of their central importance as a research resource. Our results showed that amplification efficiency was unusually low for this multilocus nuclear marker, which probably reflects the DNA quality of extracted cultures. In successfully sequenced samples, the ITS barcode was able to provide clear species demarcations and could identify 93% of strains to a known species and of these, 32 strains showed evidence of true cryptic species, revealing considerable hidden biodiversity. Another 21 strains were shown to be mis-identified.

## Results

### Overall efficiency

**Figure 1:**
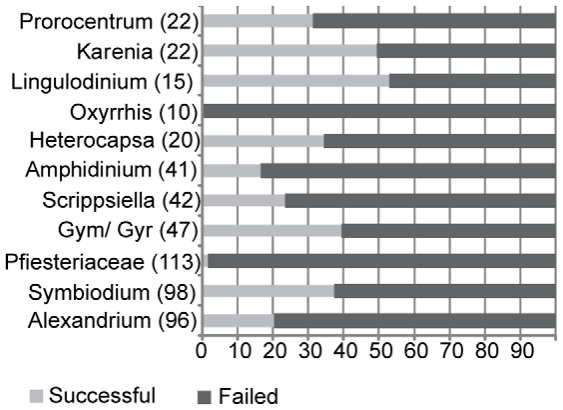
Proportion of successfully barcoded strains in our dataset for selected taxa. Numbers on X-axis are percentages. Gym/Gyr denote strains called *Gymnodinium* or *Gyrodinium*. 335 sequences failed at the amplification step (65%), 132 failed at the sequence stage whilst 47 (8%) failed due to the presence of paralogues or contaminants. The Pfiesteriaceae and *Alexandrium* taxa were proportionately worse at amplification compared to other genera.

We collected 669 dinoflagellate strains from 10 private and public culture collections and were able to obtain amplicons from 47% of these samples. After eliminating low-quality and failed sequences, we were left with 151 ITS barcode sequences from our culture collection strains, plus 242 ITS sequences from Genbank, a total of 393 ITS barcodes from 78 identified species (including species from the *Symbiodinium* complex, where we counted a species as a strain that corresponded to its smallest identified sub-clade type). By comparison, 266 COI barcodes were successfully generated from the same number of strains [11]. Only 77 strains shared both an ITS and a COI-barcode from our earlier study [11]. We compared our results to three other studies (Table S2) using taxa that were common to at least three of the studies. As different taxa were used in these respective studies, this restricted this comparison to only five genera, 15 species and 1 *Symbiodinium* group. Similar mean intra and interspecies pairwise distance (PWD) variation was found for this study (A) and that of Litaker and colleagues [20] (B) except for *Karenia* and *Prorocentrum* that likely reflects differences in the number of sequences used and the inclusion of more diverse *Prorocentrum* in this study, which has a deep –lineage split. Mitochondrial markers, *COB* (C) and COI (D) also showed similar levels of interspecies variation, except for *Symbiodinium* probably because of a large discrepancy in sequences analysed, and the different way in which this genus was classified. Intraspecies PWD between COI and *COB* were similar (varying between 0 and 1.7) in the 3 species common to both studies, although the dataset is too small to make significant comparisons. The ratio of mean inter-species versus intra-species PWD was 34 and 74 for ITS study A and B respectively, confirming a large barcoding gap between and within species. By contrast, the mean intra- to inter-species ratio was much smaller for C and D, at 10 and 1 respectively, excluding *Symbiodinium*, with *COB* showing an average barcode gap similar to animals [28]. The barcoding success rate varied widely between genera, from a low of 2% to a maximum of 53% (mean 35%), which suggests that the technical difficulties with universal ITS primers are still too extreme for it to function as a general barcoding marker for dinoflagellates. Figure 1 summarises the main representatives in the ITS database, which was heavily biased for some of the larger and more complex assemblages: *Symbiodinium, Alexandrium* and members of Pfiesteriaceae comprised two-thirds of the total taxa. Causes of failure were amplification failure (64.67%, 335 strains), failed sequencing reactions of amplified strains (26.25%, 136 sequences) and poor sequence quality (9.07%, 47 strains). *Pfiesteria* and *Oxyrrhis* were particularly poor, with almost no success, whereas *Lingulodinium*, *Symbiodinium* and *Gymnodinium*, *Gyrodinium* and *Karenia* together were above average. Twenty percent of *Alexandrium* strains were successfully barcoded, a low efficiency compared to other genera.

**Figure 2:**
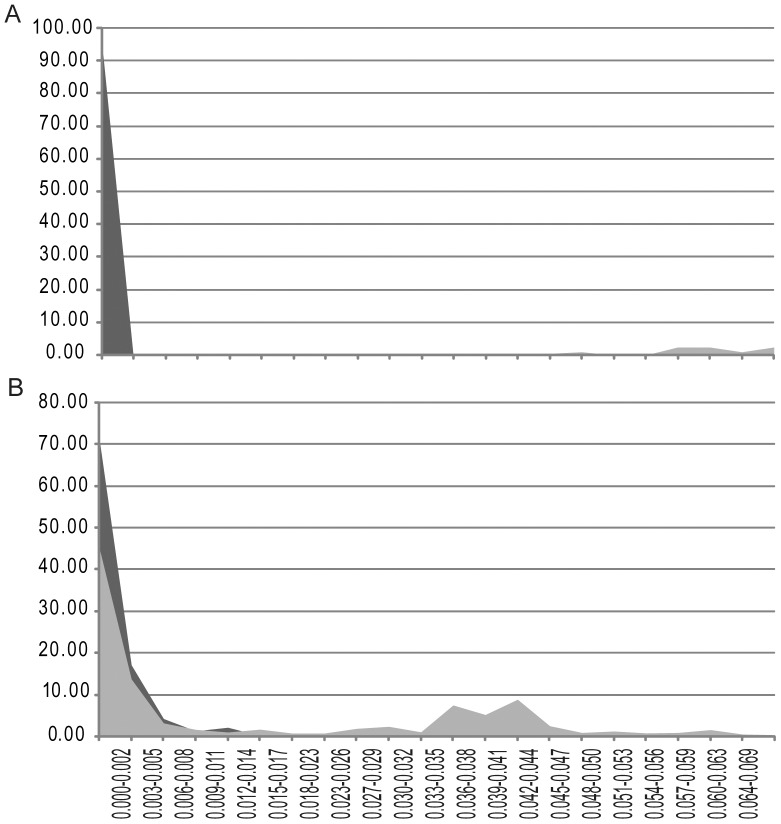
Comparison of ITS versus COI DNA barcodes in species–level identification. Panel A and B refer to ITS and COI respectively. Dark grey shading indicates intraspecific distances whereas light grey interspecific distances. Y axis shows percentage of named species and genera that fall into pairwise distance categories (X-axis). Both A and B share same X-axis. Although both ITS and CO1 barcodes fall within the 0–0.02% range, note how ITS has a sizeable gap in genetic distance within species compared to between species, that is lacking for COI marker. In this study 2% or less PWD between strains was used as a species cut off, which encompassed 94% of strains. Abbreviations: Sym: *Symbiodinium* and Sym gp. A? refers to unknown group A *Symbiodinium* sp.; Karl.: *Karlodinium*; K. ven.: *Karlodinium veneficum*; C.sp.: *Cryptoperidiniopsis* sp.; Scr.: *Scrippsiella*; S.troch.: *Scrippsiella trochoidea*.

**Figure 3:**
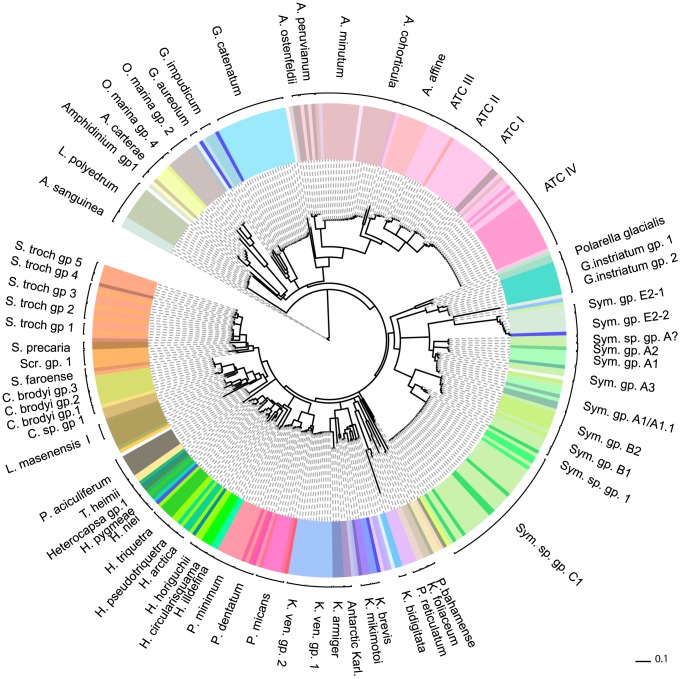
Neighbour-joining phylogenetic analysis of ITS DNA barcodes for all dinoflagellates from culture collections in this study and from GenBank. Using uncorrected p-distances. Most species could be accurately identified with ITS which showed cryptic speciation in *Scrippsiella*, *Heterocapsa*, *Oxyrrhis* and *Karlodinium*. Strain labels were removed for clarity but are available in Figure S1 and also listed in Table S1. Abbreviations: S. sp. : *Scrippsiella* species; Sym: *Symbiodinium*. Brackets represent species groups as identified using criteria described in methods and results. GB indicates a genbank deposited strain.

For those strains where an ITS marker was available, we found a high degree of correlation between species names and their uncorrected, (PWD) scores to each other, (see Fig. 2, Table S2). Our analyses show that ITS has a well-defined (PWD) gap that separated strains within a species (94% conspecific strains in our comparison had a PWD between 0–2%) compared to strains between species (see Fig. 2, panel A). By comparison, COI lacked a clear barcode gap (Fig. 2, panel B). There was a large range in genetic variation between species (4.7–41.5%, mean = 28.7%), with the greatest interspecies variation observed in *Symbiodinium* clade E and *Peridinium*. Six conspecific strains, belonging to four species (*Heterocapsa pygmaea, Peridinium cinctum, Protoperidinium reticulatum, Gyrodinium instriatum*) showed an intermediate level of intraspecies PWD values (between 3.6–4.3%) - levels higher than within species but less than within genus. We therefore used the 2.0% PWD value as a conservative cut-off value to identify a species (detailed in Table S1). These distances were mapped onto the clades of an ITS-barcode neighbour-joining tree of all strains (see Fig. 3, Fig. S1), and specifically the *Gymnodiniales* (Fig. S2), *Heterocapsa* (Fig. S3), *Symbiodinium* (Fig. S4) and *Alexandrium* (Fig. S5). Formerly unresolved genera such as *Lingulodinium* and *Protoceratium* and species within *Alexandrium* could be clearly identified as separate genera and species/genotypes, even in cases where taxa were identical using COI barcodes [11]. In other cases, strain names were either reconfirmed or renamed based on their clustering with known species. If two or more strains clustered with strains that had no species name, the strains were named after their given genera and then a group number. 93% of these strains could be identified to a known species by barcoding, and this figure increased to 96% when new ITS- barcode species groups (without a formal species name) were included. Four percent of strains (belonging to the genera *Scrippsiella*, *Symbiodinium* and *Heterocapsa*) turned out be identical to at least one other strain based on their ITS sequences but not to any known species, whilst 21 strains had mismatch between strain name and its identity. More interestingly, 8.3% of strains identified by barcoding showed evidence of true cryptic species, excluding known species complexes (see materials and methods) revealing a hidden biodiversity of dinoflagellate species.

Assigning species-level identities to the genus *Symbiodinium* was problematic because species names have largely been replaced by phylogenetic clade and sub-clade type identities [29]. *Symbiodinium* clades only partially correspond to the sixteen species and subspecies designations assigned to the genus [5], [9], [30]–[32]. Each clade is further divided into “types” [10] that represent strains with a unique allele. To assign species-level identities to strains in a manner that corresponds to other dinoflagellate barcode species designations, the same 2% cut off was used to cluster strains which were given clade or subclade categories.

The harmful algae, *Alexandrium*, formed a significant proportion of our database, many of which belonged to the Alexandrium Tamarense Complex (ATC) that comprises *A. tamarense*, *A. catenella* and *A. fundyense*. Previously thought to be three separate species, it has been shown that they are overlapping morphotypes of the same species [3], [33]. Instead, ATC genotypes appear to group into six geographical regions [34]. *A tamarense* morphotypes are cosmopolitan whereas *A. catenella* are found in North America (NA) and Temperate Asia (TA) [34]. Strains from these areas can be toxic. Four main genetic ATC groups based on D1–D2 Large Ribosomal Subunit (LSU) have also been identified [33]. Several of these strains analysed by LSU were also included here, and additional common strains could be found in database collections, enabling us to cross-reference ITS barcode groups to ATC groups I, II, III and IV and compare ATC groups to strains identified as *A. tamarense* and *A. catenella*. Overall six *Alexandrium* strains did not match their species name and a further 47 ATC strains have now been categorized into their genotype groups (see Fig. 3, Table S1). Linking two common strains with those identified by the small ribosomal subunit and the whole ITS region has enabled us to assign strains to ATC geographic genotypes [34], [35]. ATC I corresponds to North America and Japan and in our dataset consists of Asian isolates except for strain MDQ1096, isolated from Argentina. ATC II is a Mediterranean clade consisting of only Mediterranean isolates except for OF935-AT6 from Japan. ATC group III was originally Western European with strains from the English Channel and Mediterranean but also included strains from China. Two strain synonyms of CCMP 115 in this study also belonged to ATC III, although were non-identical, possibly indicating a heterogeneous culture or contamination. Finally, ATC group IV corresponds to the temperate Asian clade, consisting of all *A. catenella* strains collected from the Mediterranean [35] plus *A. tamarense* strains from Asia. These strains were part of a study to show ATC clade IV had invaded the Mediterranean. CU-15 and an *A. cohorticula* strain had identical ITS sequences, the only 2 representatives of the Tropical Asian strain group.

Identification of the polyphyletic *Amphidinium* genera was also successfully achieved. This group could not be amplified using COI marker, but ITS correctly identified members of *Amphidinium sensu stricto* group [36]. Five strains of *A. carterae* were matched their labels, but UTEX 1946 *A. rhynchocephalum* (a synonym of *A. operculatum* [37]) and *A. massartii* (NEPCC 802) were found to be identical and might indicate a misidentification or contamination in one of these strains.

The next largest genus investigated here was *Symbiodinium*. This is a challenging group to investigate not only because of its diversity, but because obtaining monotypic cultures is difficult to obtain directly from their host [38] so cultures very often contain more than one genotype. We compared previously identified and unidentified strains in our ITS database, and were able to place thirty-three unassigned strains into known clades. These corresponded to barcode species groups. Only three strains remained unidentified-two of these belonged to an unknown group. *Symbiodinium* clade A [39] was subdivided into five sub-types, A1, A1/1.1, A2 and A3 and an unknown group A, and clade B into B1 and B2 based on strains previously identified by LaJeunesse *et al.* [9], [40] and Santos *et al.* [41], [42]. Some strains had two additional gene identifications, a COI barcode group [11] and/or a genotype derived from the hypervariable region within Domain V of chloroplast 23S gene (Cp23S-rDNA) [42]. The largest *Symbiodinium* cluster was subclade C1, with 35 strains and no cryptic species in our sample set. ITS barcoding placed CCMP 2466 into group C, which was ambiguously assigned C or F in our previous COI barcoding study, as clade F could not be distinguished from clade C [11] (see Table S1).

For clade A and its subclades, strains belonging to ITS barcode groups A1 and A1/1.1, and one of the three A3 strains, CCMP 2592, corresponded well with the unresolved COI barcode group Ax described by Stern *et al.* 2010 [11]. Other ITS barcode groups in this study (B, C, E) were also consistent with COI barcode categories. Correspondence between ITS barcodes and Cp23S-rDNA genotypes [42] was also very good, overall. The one representative of Cp23S-rDNA genotype A198 corresponded to ITS group A3. ITS barcode groups A1/1.1 corresponded to Cp23S-rDNA genotype A194 with two exceptions: strain MAC-K 20.1.6 (Cp genotype A188) strain also grouped with A1/1.1. By contrast, a second Cp A194 strain, MAC-04-218, failed to cluster with the A1/1.1 or any other *Symbiodinium* group in our study. Our previous COI barcode study [11] assigned this strain to clade A3. These anomalies within A194 can either be interpreted as strain misidentification, or a partial overlap between ITS clades and Cp23S-rDNA genotypes.

All strains belonging to ITS group B were subdivided by their PWD into 2 groups. Strains Pk 13 SD1, Gv5.6c, Pk706.16-SCI, Mf 01.05b01 and Mf 01.05b02 belonged to group B1, most similar to clade B1whilst strains Mf 10.14b.01, K 17.1.3, K 17.1.3.6 and K 17.1.3.9 formed group B2. Group B2 was so named because two strains showed species level identity to the third member previously assigned to clade B2 [9], [43]. However strains 579 and 571, that were genotyped as B19 and B25 using the ITS2 marker, also belonged to group B2. These strains shared features of both clades B1 and B2 but also had unique single nucleotide polymorphisms in the ITS2 region distinct from B1 and B2.Thus, the PWD cut-off method used here has grouped several distinct genotypes together and may be less sensitive to detect different genotypes. We also found strain 201 (clade F) matched group B2 but have attributed this mismatch to mixed culture. The one representative of Cp group B224 appeared to be a borderline group B strain.

### Paralogues

Because ITS paralogues have been reported as a confounding factor in measuring species diversity [20], we investigated their influence on species detection by calculating pairwise distances between 127 clonal variants of ITS from 22 different dinoflagellate strains (see Table 1) deposited in GenBank. For example, *G. instriatum* forms two groups, consisting of directly sequenced strains and a second group that contains several clonal variants, which may represent a different paralogue of ITS. Clonal variation never exceeded the 2% species cut-off in this small sample set, except for clones of *Symbiodinium* type E2 *sensu* [9], discussed below. Between 2–22 (mean 5.7) clones per strain were examined. This is a modest sample set but it did contain *Symbiodinium* and *Prorocentrum* that exhibited higher clonal genetic distances that indicates that PWDs arising from paralogues are smaller than the species-level cut off of 2%. However, many of these strains are cultures that will have lower genetic variation that, in turn, may artificially reduce paralogue variation even further. Additionally, the intragenomic variation from a larger dataset of environmental as well as cultured strains, may exceed this value [26], [27].

**Table 1:** Summary of Clonal strain variation of dinoflagellate ITS sequences from public databases.

Species	Clone name	Genbank accession	Max	Min	Mean	S.D	N
*Gyrodinium instriatum*	clone 2,9,15,18	AJ534383, AJ534386-8	0.008	0.000	0.003	0.005	5
*Karlodinium micrum*	GgaITSC	AF352365-6, AF352368	0.014	0.000	0.010	0.007	3
*Karenia brevis*	GbrITSC	AF352368-9	0.004	0.004	0.004	0.000	2
*Cryptoperidiniopsis* sp.	A5 CspA5	AF352355-8	0.003	0.000	0.002	0.002	3
*Pseudopfiesteria shumwayae*	PshVIMS1049ITSC	AF352341-4	0.008	0.000	0.005	0.003	5
*Pseudopfiesteria shumwayae*	PshCellNS	AF352338-40	0.008	0.003	0.005	0.002	3
*Pfiesteria piscicida*	PpiCellM	AF352333, AF352337	0.003	0.000	0.002	0.002	2
*Prorocentrum minimum*	PmiITSC	AF352370-1	0.016	0.016	0.016	0.000	2
*Heterocapsa triquetra*	HtrITSC	AF352363-4	0.002	0.002	0.002	0.000	2
*Pyrodinium bahamense*	PBSA	AF051366, AF145225	0.000	0.000	0.000	0.000	2
*Gymnodinium* sp.	NVA/RUS/2008	HQ270472-3	0.003	0.003	0.003	0.000	2
*Symbiodinium* sp.	kokubu	AB190265-72	0.019	0.000	0.010	0.005	8
*Symbiodinium* sp.	Amami clone 1	AB207197-204	0.011	0.003	0.007	0.003	7
*Symbiodinium* sp.	Amami clone 5	AB207208-9	0.005	0.005	0.005	0.000	2
*Symbiodinium* sp.	Amami clone 4	AB207205-7	0.010	0.002	0.006	0.004	3
*Symbiodinium* sp.	Amami clone 3	AB207193-5	0.019	0.010	0.016	0.005	3
*Symbiodinium* sp. *clade C*	FF	AB294585, AB294604-9	0.006	0.000	0.002	0.002	7
*Symbiodinium* sp. *clade C*	Fu-02	AB294593-603	0.004	0.000	0.002	0.001	11
*Symbiodinium* sp. *clade C*	FU-21	AB294640-661	0.009	0.000	0.003	0.003	21
*Symbiodinium* sp. *clade C*	F1-18	AB294610-22	0.019	0.000	0.009	0.005	13
*Symbiodinium* sp. *clade C*	Cc-19	AB294623-26	0.007	0.001	0.001	0.002	4
*Symbiodinium* sp. *type E2*	clone E2	EU079408-EU079424	0.062	0.000	0.027	0.022	17

Numbers relate to PWD maximum (Max), minimum (M) and mean values.

### Strain Synonyms

Strain misidentification is a serious issue in culture collections [44], and we therefore included as many strain synonyms as possible to detect cases of misidentification, which may arise for a number of reasons: mislabeling, culture contamination, but may also arise if a culture started with 2 cryptic species or have undergone sexual recombination in culture. This study was able to highlight that four strains were not identical to their respective strain synonyms cultured elsewhere and a further 2 sequences (*Heterocapsa arctica*) of the same culture were not identical (see Table 2). The differences in *Gyrodinium instriatum* strains CCMP 431 and NEPCC 796, maybe a mislabeling issue as CCMP 431 was identical to a second *G. instriatum* strain, whereas NEPCC 796 was identical to another *G. dorsum* strain. For 2 species, *Karenia mikimotoi* and *Heterocapsa arctica*, the sequences were not identical, but within species-OTU boundaries at PWD = 0.6%. Cryptic speciation has been observed for *K. mikimotoi* [45] and may be an explanation for the variance observed in *H. arctica*.

**Table 2:** Strain synonym variation in dinoflagellate ITS barcodes.

Strain ITS barcode identity	BOLD label/Genbank accession	Culture Collection	Strain synonym	DNA distance
*Akashiwo sanguinea*	DINO1219-08	NEPCC 885	CCMP 1837	0.000
*Akashiwo sanguinea*	DQ779988	CCMP 1837	NEPCC 885	0.000
*Alexandrium affine*	DINO1173-08	NEPCC 667	CCMP112	0.000
*Alexandrium affine*	AY831409	CCMP 112	NEPCC 667	0.000
ATC group III	DINO1077-08	NEPCC 802	UTEX 1946	0.000
ATC group III	DINO779-07	UTEX 1946	NEPCC 802	0.000
*Amphidinium* sp. group 1	DINO1188-08	CCMP 115	NEPCC 183, PLY 173	0.000
*Amphidinium* sp. group 1	DINO1071-08	NEPCC 183	CCMP115, PLY173	0.000
*Gyrodinium instriatum* group 1	DINO1175-08	NEPCC 796	CCMP 431	**0.037**
*Gyrodinium instriatum* group 1	DINO923-08	CCMP 431	NEPCC 796	**0.037**
*Heterocapsa arctica*	DINO1192-08	CCMP 445	CCMP 445	**0.006**
*Heterocapsa arctica*	AB084095	CCMP 445	CCMP 445	**0.006**
*Heterocapsa illdefina*	DINO1176-08	CCMP 446	CCMP 446	**0.005**
*Heterocapsa illdefina*	AB084092	CCMP 446	CCMP 446	**0.005**
*Karenia mikimotoi*	DINO916-08	CCMP 430	NEPCC 665	**0.006**
*Karenia mikimotoi*	DINO766-07	NEPCC 665	CCMP430	**0.006**
*Kryptoperidinium foliaceum*	DINOB781-08	CS-37	UTEX 1688	0.000
*Kryptoperidinium foliaceum*	DINO409-07	UTEX 1688	CS-37	0.000
*Symbiodinium* sp. clade E2-1	DINO356-07	NEPCC 737	CCMP 421	**0.003–0.057, mean 0.003**
*Symbiodinium* sp. clade E2-2	DINO1227-08	NEPCC 795	CCMP421	**0–0.066, mean 0.005**
*Symbiodinium* sp. clade E2-2	DINO979-08	NEPCC 860	CCMP421	**0.01–0.078, mean 0.013**
*Symbiodinium* sp. clade E2-2	DINO929-08	AC561	CCMP421	**0.003–0.069, mean 0.01**
*Symbiodinium* sp. clade E2-2	AY160123	G15	CCMP 421	**0.008–0.068, mean 0.008**

Bold face indicates PWD values higher than species-barcode cut off of 2%. N = number of sequences used, S.D = standard deviation.

One anomaly we observed was the high diversity of directly sequenced strain synonyms of CCMP 421 from New Zealand, which comprised our entire *Symbiodinium* type E2 dataset, with one exception, AC 561, (see Table 2). As this strain had previously been reported to contain pseudogenes or paralogous ITS sequences [11], [27] that may confound analysis, we also compared their cloned products with our directly sequenced ones. One clone, E2 2092 (GenBank accession EU074911), was exceptionally diverse with 6.8% median difference to other strains. Two sub-groups were identified for *Symbiodinium* clade E. Group E2-1 contained one CCMP 421 clone, and our directly sequenced strain synonym of CCMP 421 (NEPCC 737). Group E2-2 consisted of NEPCC 860 and NEPCC 795 (two directly sequenced strain synonyms of CCMP 421), a cloned ITS sequence of an independent Chinese strain called G15, two further clones of CCCMP 421 plus directly sequenced strain AC 561 recently re-assigned to *Symbiodinium* clade E by COI barcode analysis [11]. Comparing our directly sequenced strains in both groups E2-2 and E2-1 against the 5.8S ribosomal DNA (part of the ITS marker) of CCMP 421 [27] showed complete sequence identity, with the exception of a C instead of G for AC 561 at position 133 of the 5.8S rDNA marker, and a T instead of a C in NEPCC 737. Neither of these positions corresponded to sites reported to have high substitution rates.

### Strain identification anomalies

**Figure 4:**
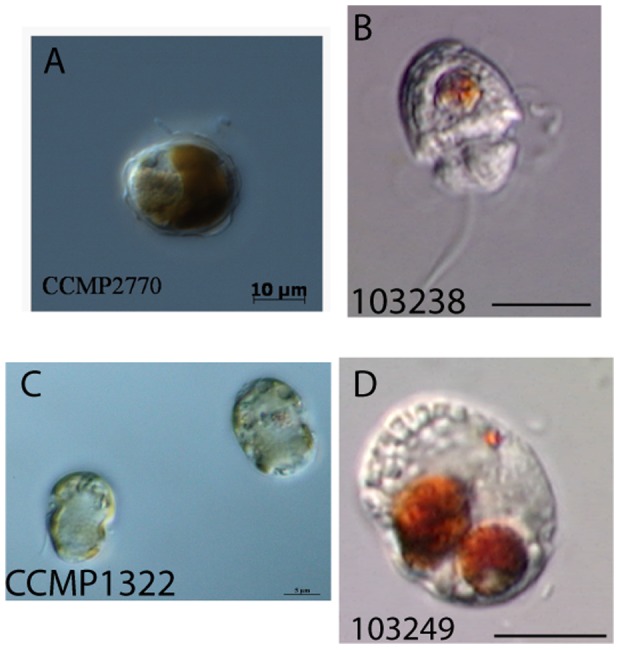
Light Micrographs of *Heterocapsa* group 1, CCMP1322 (A), CCMP2770 (B) and 103238 (C), revealing different morphologies. This genus showed one of the highest levels of strain name incongruities. 103238 and a third strain 103248 (D), were both putatively identified as *Katodinium asymmetricum* but ITS-barcoding showed the latter was unrelated to this or any other dinoflagellate studied here. Note pigments of 103238 and 103248 belong to cryptophytes food and these strains are heterotrophic.

Most strains in this category belong to morphologically identical or poorly characterized species, namely *Alexandrium, Gyrodinium*, *Prorocentrum*, *Symbiodinium* and *Heterocapsa*. The ITS barcode was able to differentiate *Heterocapsa triquetra* and *Heterocapsa pseudotriquetra* [46], however two other *Heterocapsa* species showed mismatches whose identity was further confused by multiple name synonyms. *Heterocapsa pygmeae* (CCMP 1322) [46] was identical to CCMP 2770 called *Glenodinium hallii*, which switched to *Cachonina hallii* and now *Heterocapsa hallii*. *Heterocapsa hallii* is in turn sometimes recognized as a heterotypic synonym of *Heterocapsa illdefina* [47]. These strains are unlikely to be *H. illdefina*, however, as they are different from two *H. illdefina* strains (CCMP 446) identified by an earlier taxonomic study [46]. Both CCMP 1322 and CCMP 2770 also showed species level identity to another heterotrophic dinoflagellate (103238, see Fig. 4), putatively named *Katodinium asymmetricum*. All three dinoflagellate strains were morphologically different by light microscopy. To complicate matters further, *K. asymmetricum* and a third *Heterocapsa* species, *Heterocapsa rotundata*, have identical thecal plate morphology, a defining feature of this genus [48]. *Heterocapsa* species are small and so there is a possibility that these barcodes are from one or several contaminated cultures, although 103238 was sequenced twice from two different DNA extractions. Nevertheless, there is also taxonomic similarity between *Heterocapsa* and *Katodinium* too [49]. Given the ambiguity in the identity of these three dinoflagellates, we called these strains *Heterocapsa* group 1.

Another anomaly within the genus *Heterocapsa* was the identity of “*Gymnodinium* sp.” CCMP 424, that showed species-level identity to *Heterocapsa niei* strain CS-36. The COI barcode of strain CS-36 showed it belonged to a *Symbiodinium* group [11]. However, *Heterocapsa* was one of the genera for which COI barcode amplification failed, so comparisons to known *Heterocapsa* species was not possible. This identity was confirmed by comparing it to an independent GenBank sequence of CCMP 424 (EF492492) and by sequencing the partial sequence of the small ribosomal subunit (SSU) of this strain, which showed closest identity to a *Heterocapsa* sp.

### Cryptic Variation and Species Complexes

ITS barcodes revealing cryptic variation that could represent new species were given a genus name and group number. Most cryptic variation was found in *Gyrodinium instriatum* (2 groups), *Karlodium* (2 groups) and other *Cryptoperidiniopsis* sp. not belonging to *C. brodyi*.

*Gyrodinium instriatum* strains were found to correspond to two distinct groups. Group 1 isolates were derived from two distinct environments in Portugal, whereas group 2 isolates were all from the same region of Guangdong province in China, indicating a biogeographical separation of potentially two species. The South Korean strain of *Gymnodinium aureoleum*, DQ779991 (GrAr01), now identified as *Gyrodinium aureoleum* [46], was not identical to a second *G. aureoleum* strain (SWA 16 from Namibia). Morphologically this species is very similar to *Karenia mikimotoi* [50]. One of these strains may belong to *K. mikimotoi* or else may be a cryptic species, but indicates the difficulties identifying gymnodinoid species.

Considerable variation was also found in known species complexes including ATC and *Symbiodinium* clades (both described above), *Oxyrrhis marina*, *Cryptoperidiniopsis brodyi*, *Luciella mansenensis* and *Scrippsiella trochoidea*. Five subgroups were found within the *Scrippsiella trochoidea* species complex from 15 *S.trochoidea* and 4 *Scrippsiella* species [11], [22], [51] One of these groups, *S. trochoidea* group 3, had one strain in common with our COI study [11], enabling us to assign four more strains that belong to *S. trochoidea* group III. However, two additional strains could not be placed into any subgroup, and may represent a novel *S. trochoidea* group. Four *Scrippsiella* strains, belonging to STR1 clade in a study by Steidinger and colleagues [52], were included in this study. These same strains formed *S. trochoidea* barcode-groups I and II. In their study NIES-369 belonged to clade STR2 which corresponds to *S. trochoidea* barcode group 3 in this study. All five of these strains were reported highly divergent and widely distributed from shelf localities, belonging to a phylogenetic group mostly with spiny cysts [52].

*Cryptoperidiniopsis* [52] shares a similar morphology, behaviour, and habitat with *Pfiesteria* and *Luciella* [53]. Members of these species complex are accordingly difficult to identify morphologically and their complex life-cycles also make identification of species challenging but important. ITS barcodes could distinguish all members of these genera. Eight *C. brodyi* strains from Australia fell into three subgroups that were separated from *C. brodyi* strains from USA. At least 2 more unconfirmed *Cryptoperidiniopsis* species could also belong to *C. brodyi* (H/V14 and PLO21) a resolution is much greater than that achieved previously with rDNA, which could only resolve these strains into two genotypes and has also been reported to give false results [54]. Likewise, ITS reliably confirmed two out of four *Luciella* ribotypes [53], and revealed a new ribotype I strain, CCMP 1955. NC Lucy-V27 was unconfirmed because this strain was separate from other *Luciella* sp. and was not described in original *Luciella sp.* study. COI barcoding identified three subgroups of *Luciella*, but unfortunately these groupings could not be cross-referenced as there were no strain sequenced for both COI and ITS barcodes. None of *Luciella* strains matched “Shepherds Crook” (AY590479) or Jeong2006-1 strains, which are distinct, as reported previously [55], [56].

All four *Oxyrrhis marina* clades identified by Lowe and colleagues [57] were also recovered in our re-analysis based on GenBank-deposited ITS sequences and our 2% cut off value. Lowe *et al.* [57] proposed at least two species groups, given the high diversity of this genus.

## Discussion

Living repositories of collected algae are used for research and aquaculture, and culture collections need to ensure accuracy against inevitable contamination, mislabeling, and confusion among multiple strains for a cultured species. DNA barcoding provides a means for identifying species using a common measure of species differentiation. The ideal DNA barcode marker is one that can both be acquired from all target taxa and can distinguish them. We have shown by extensive species sampling of previously identified dinoflagellates from culture collections that the ITS marker has the ability to successfully identify 96% of strains tested at a 2% species cut-off level, including three that had no genus identity. A comparison of this study to previous dinoflagellate barcoding studies [11], [12], [20], [58], [59], shown in Table S2, was limited, as different species and taxon definitions are used. However COI and COB appear to be broadly comparable in terms of species range and variation, although the barcoding gap was wider for COB. For ITS, this study was in agreement with earlier findings for this marker [20]. Our species-level cut off in dinoflagellates is slightly lower than the species-level cut-off value of 4% (p = 0.04) observed by Litaker and colleagues [20] but similar to the value for which we observed interspecies PWD, at 4.9%. We used a conservative cut-off of 2% as some of the conspecific strains in our study had higher PWD values, between 3.6–9.4%, such as *Prorocentrum* and *Heterocapsa*. These values reflect cryptic diversity (*Heterocapsa*) or deep lineage split between species in a genus (*Prorocentrum*) [60]–[62]. Using a genus-specific barcoding approach may lead to inaccurate species assignments and would be unlikely to work for the vast majority of unknown dinoflagellates collected from environmental surveys.

As predicted by culture collection managers [44], mismatches were identified and we found 21 strains that belonged to a different species, excluding potential clonal variants, cryptic species and species complexes (see Table S1). Whilst ITS had a lower amplification success rate than COI overall, members of nearly all genera could be amplified and successfully identified (e.g. *Lingulodinium*, *Protoceratium* and ATC). By contrast, COI primers used in our previous study [11] had a non-random pattern of failure, such that some genera (e.g *Amphidinium, Heterocapsa, Oxyrrhis*) were never successfully amplified. This suggests that ITS failures might be sample-specific whereas COI failures are due to intrinsic factors. Overall, the ITS marker has a well-defined range informative of species-level diversity which does not overlap with the observed genus-specific range. A good barcode marker will show a greater genetic distance between different species, compared to strains belonging to the same species. This is known as a barcode gap, and was found using the ITS marker both in this and a previous study [20]. *COB* is also a potentially useful marker, and demonstrated a suitable interspecies genetic distance that was 10 times larger than the intraspecies distance [12], although further investigation is required with more strains to confirm this gap remains. Such is the major drawback of COI, which lacks such an interspecies barcode gap that could lead to false positive identification. Additionally, this constrains the number of species that can be identified and leads to an underestimation of real biodiversity because of the low cut-off values applied. For the case of COI, only 72% of species could be identified at a cut-off level of 0.2% compared to 95% at 2% level with ITS [11]. One example of the success of the ITS, was the ATC group. Our study confirmed geographical groupings reported in previous studies [34], [35], [63], [64] using ITS, SSU and LSU. Additionally, ITS and the D1–D2 variable region of LSU were shown to be congruent with those found by Lilly and colleagues [33], allowing us to link DNA barcodes with more in-depth taxonomic studies. Here, additional strains from toxic ribotypes were identified: Tropical Asian, Temperate Asian and North American. New strain localities within groups were found, for example an Argentinean strain in North America group. These ITS barcodes will permit better comparisons from more variable natural strains. By contrast, genotypes of ATC were unresolvable using the CO1 barcode marker [11].

The major problem that we identified with ITS as a barcode marker was the sequencing efficiency. We observed a success rate of 50% with ITS compared with 66% for COI. This was lower than expected for a multi-copy marker. We attribute this to poor quality DNA extraction, which is a common problem in dinoflagellates and other algal groups. We suffered a similar problem using COI marker, which required two rounds of PCR to improve amplification success [11]. Most cultures were extracted from restricted volumes and many were sent by courier to us. A combination of these could lead to insufficient concentration of DNA. Some genera amplified better than others. The poor performances of Pfiesteriaceae and *Alexandrium* strains present in high numbers in our dataset skewed the success rates. However, poor sequencing success rates may also be due to many factors including non-axenic cultures (in combination with eukaryotic ITS primers), robustness in transit, presence of theca leading to suboptimal DNA extraction success.

The predicted problem of ITS paralogues resulting in double peaks was relatively minor in this study but is probably a larger problem for highly diverse genera. In environmental samples, this would pose a significant problem as paralogue variation in natural populations could be higher. Aligning ITS is challenging, but the use of a large dataset of taxa from members of the same genus can result in better alignments where informative sites could be identified at every taxon level, making paralagous copies easier to spot. Our study showed that average PWD between detected paralogous copies of the ITS marker was lower than the PWD cut-off values which we applied to distinguish species, although the sample set examined was small. Likewise Litaker and colleagues [20] found intragenomic distances from cloned paralogues of a strain was at least half that of the distance between two species. They also demonstrated that strains would possess a common clonal variant that was more likely to be recovered than the rarer variants. Intragenomic variation is predicted to be lower than intergenomic variation in eukaryotes including dinoflagellates [65]–[69] due to a genetic mechanism called concerted evolution within a genome. In the case of dinoflagellates there are numerous gene paralogues including COI that make the situation more complex. However, our study showed that *Symbiodinium* clade E did exceed the 2% species cut-off, confirming findings of earlier studies of this strain [11], [27], possibly as a result of heterogeneous culture or genetic change during culturing. Our dataset had a high paralogous variation in Table 1 because it contained a large proportion of *Symbiodinium* species which are diverse, a process possibly facilitated by the symbiotic and/or free-living nature of many strains within clades A, B, C, and E. It is likely that different dinoflagellate lineages exhibit varying diversity levels, of which *Symbiodinium* is probably an extreme example. The ITS has been extensively used to classify the highly diverse *Symbiodinium* genus and a recent in-depth study showed that the ITS2 marker was ideal for distinguishing ecologically distinct *Symbiodinium* species based on multi-gene comparison [25]. We found good correspondence with ITS2 types and barcode groups. However, our study is likely to underestimate *Symbiodinium* diversity as we used the entire ITS marker instead of ITS2 region, which resulted in clustering more than one *Symbiodinium* type into a single barcode group.

Recent studies have revealed how paralogues and chimeras in environmental studies can over-estimate or confound phylogenetic analyses [26], [70], [71]. Thus the applicability of ITS as a single universal marker is questionable given the issues of paralogues, particularly in the *Symbiodinium* group [27], and the variable evolutionary divergence of different dinoflagellate genera, especially when applied to heterogeneous environmental samples (water or sediment). ITS-barcoding may only be useful for taxon-specific studies, unless new methods to distinguish intragenomic variability are developed. Whilst direct sequencing has the advantages of detecting the dominant intragenomic type, cloning can also be applied to distinguish paralogues, although the latter method can confound phylogenetic analyses through the production of chimeras in environmental samples [71]. To circumvent problems of paralogues, we propose a cloning or nested strategy plus the use of a non-nuclear secondary marker for environmental studies. A recent study has suggested the psbA gene [71], which was also proposed as a barcode marker [72]. The chloroplast marker (Cp23S-rDNA) and COI are also effective [11], [42] but may not always show complete correspondence to each other and to ITS barcodes for some *Symbiodinium* clades. *COB* may be a suitable candidate [12] but has not been tested at depth. The development or improvement of dinoflagellate-specific primers may improve dinoflagellate-specific amplification success.

Our results did show genetic differences within strains. Aside from obvious possibility of strain contamination and sequencing errors, recent studies have highlighted genetic instability in long-term cultured strains [73]. For culture collections, DNA barcoding is therefore an important tool to measure genetic stability of their strains. Lowe and colleagues [57] carried out an environmental diversity study of *Oxyrrhis marina* strains, including cultured strains that could be separated into four clades that corresponded well with our barcode-species groups. However, in their study, clade 4 comprised only cultured strains that could not be matched to any of their environmental samples. This may be due to insufficient sampling but raises the possibility that indicate genetic instability in long-term cultures.

ITS barcoding proved useful in identifying cryptic species and possible speciation events in strains of *Pfiesteria* and *Luciella*, and the related *Cryptoperidiniopsis*, where cryptic speciation and biogeographical separation are factors. This variation is unlikely to be caused by paralogues, as the Pfiesteriaceae ITS clones had much lower variation (0.2–0.5%). Ten separate barcode-species could be distinguished from the five original taxa. It is likely that there are many more cryptic species in this family and that the ITS would be a good marker to distinguish species of this harmful dinoflagellate group.

This study highlighted the need for a systematic re-examination of *Heterocapsa* as the number of incongruences within the *Heterocapsa* species was especially large, an observation also reported by Litaker and colleagues [20]. This genus is small and its plate tabulation difficult to identify so has often confused with *Gymnodinium* and *Katodinium* because its plates are so thin and the cells appear naked [49]. Accurate identification is important because some species, such as *H. circularisquama*, are harmful algal bloom species [74]. *Glenodinium* was also confusingly used to name former *Heterocapsa* and *Cachonina* species, adding another layer of complexity. Our ITS results were mostly congruent with scale morphology, a major species-diagnostic feature [46], in identifying eight species common to both studies, including *H. triquetra* and *H. pseudotriquetra*, that have the same scale morphology. Once barcode groups were established, we found that three cultured strains of *Hetercapsa* had disparate identities. For *Heterocapsa* group 1, our study highlighted confusion in both name synonyms and that of morphological versus genetic identification for the genus *Heterocapsa*. *Heterocapsa* group 1 contained three morphologically dissimilar strains with at least four possible species names, *H. pymaeae, H. rotundata*, and *Heterocapsa hallii* and *Katodinium asymmetricum* that all belonged to the same ITS-barcode group. Further studies on these strains are required to confirm their identity. Finally, the most unusual finding was that of *Gymnodinium* sp., (CCMP 424) that was identified as *Heterocapsa niei* along with strain CS-36. This result is in conflict with COI barcode results for CCMP 424, which showed genus-level similarity to the *Symbiodinium* clade A [11]. This strain was re-sequenced using ITS and SSU to confirm that the discrepancy was not the result of a PCR contamination, but the same *Heterocapsa*-like sequence was obtained. Since COI barcodes could only distinguish *Symbiodinium* to the clade level, and because some likely other fast-evolving species, (including *Heterocapsa* species) could not be acquired for COI, the placement of this strain with *Symbiodinium* is probably not an accurate representation of a genus-level relationship but rather one at a higher taxonomic level. Interestingly this strain showed 89% similarity to a cultured *Gymnodinium* strain, USA29-9, that may indicate some confusion in assigning species name, or a diverse species. Given the number of strain name changes in *Heterocapsa* group, *COB* may prove a worthy second barcode for this group, as it is easily amplified and has good resolution [12].

Overall, ITS has proved to be a suitable marker to identify a large proportion of dinoflagellate species, and is in principle applicable to all genera if the sequencing success rate observed here is due to sample quality and not some intrinsic factor. It is clear that DNA barcoding with a high resolution marker can flag taxonomic anomalies, especially in morphologically plastic taxa and in taxa that require taxonomic revision. With a considerable database, the ITS marker is a promising tool for strain quality control in culture collections, by detecting contaminations and mis-identifications. For all DNA barcoding studies with high strain numbers, the use of another marker and back-up DNA samples is recommended to reduce contamination, to identify inflated diversity due to pseudogenes and to ensure accurate identification.

## Materials and Methods

### Sample Collection

Six hundred and sixty-nine cultures or DNA samples were donated or purchased from ten public and two private culture collections, listed below with their abbreviations and also summarized in Table S1. The Culture Collection of Marine Phytoplankton, CCMP (now called National Centre for Marine Algae and Microbiota), Bigelow lab, ME, USA; UTEX, the culture collection of algae, TX, USA; the North East Pacific Culture Collection (NEPCC), that is part of the Canadian Centre for the Culture of Microorganisms (CCCM), BC, Canada; the Culture Collection of Algae and Protozoa (CCAP), UK; Roscoff Culture Collection of Marine Phytoplankton (RCC); France; Algobank Caen (AC), France; the Australian National Algae Culture Collection (CS-), Tasmania, Australia; Cawthron Institute's Culture Collection of Micro-algae (CAWD); Microbial Culture Collection at National Institute for Environmental Studies (NIES), Tsukuba, Japan. Strains donated by Hayley Skelton, formerly of North Carolina State University, NC, USA have six digit identification code, prefixed by 103. *Symbiodinium* strains donated by Mary-Alice Coffroth, University at Buffalo, USA are prefixed by MAC in Table S1 but ITS barcodes related to MAC strains are prefixed by DINO in public databases.

### DNA extraction

Typically between 1.5–15 ml of dinoflagellate cells from culture were collected by centrifugation initially at 3000 g then at 1150 g, snap frozen in liquid nitrogen and thawed three times. For one third of culture collection samples, additional grinding was performed using plastic pestle and microfuge tubes. DNA extraction was carried out using the DNeasy plant purification DNA kit (Qiagen, Mississauga, ON, Canada), following their protocol except incubating cells in lysis solution for 30 minutes instead of 10 minutes. The Masterpure Complete DNA and RNA Purification Kit (Epicentre Biotechnologies, Madison, WI, USA) was also used in about one third of cultures and for single cells, using Lysis of Fluid sample protocol followed by Precipitation of Total DNA protocol.

### PCR and Sequencing

Amplification was performed using primers ITS1 5′GGTGAACCTGAGGAAGGAT 3′ and ITS4 5′ TCCTCCGCTTATTGATATGC 3′ [75]. PCR amplification reaction was carried out on 25–100 ng of DNA using PuReTaq Ready-to-Go beads (GE Lifesciences, NJ, USA) at 94°C for 31minutes followed by 35 cycles of 94°C for 30 seconds, 47°C for 30 seconds and 72°C for 45 seconds, ending with a 72°C extension step for 7 minutes, resulting in products ranging from 500–600 bp. All culture collection ITS amplicons were sequenced directly. Single PCR products were diluted to 30 ng/µl or purified by gel extraction using the QIAquick Gel Extraction kit (Qiagen, Mississauga, ON, Canada), according to manufacturer's instructions and were either sent to Canadian Centre of DNA Barcoding, Guelph, ON for DNA sequencing or were sequenced directly using BigDye v3.1 reagents and sent to NAPS unit at University of British Columbia, BC for capillary electrophoresis. All sequences generated from this study are listed in Table S1 with Genbank accession numbers) and on the BOLD database in DAITS project at http://www.barcodinglife.org/views/projectlist.php?&.

### Sequence analysis

Sequences were manually edited using Sequencher v4.2 (Gene Codes Corporation, Ann Harbor, USA), aligned using MAFFT [76], and ambiguous sites were excluded using MacClade 4.07 [77]. ITS sequences were initially screened for obvious contamination using BLAST [78] and by correspondence with other strains of the same species. Cluster analysis of aligned sequences was performed using the neighbor joining model with uncorrected distances using PAUP* 4.0b10 [79] in order to compare PWD values between strains. Cluster analysis was visualized by ITOL web based software [80], at http://itol.embl.de/. Sequences were considered to represent the same species if they diverged by 2% or less. All PWD were calculated from a single global alignment of ITS barcodes. Cryptic species groups (i.e. newly identified groups to which no link to an existing species could be made) were labeled by species then a group number. A roman numeral system was given for those genera that belonged to a species-complex or were assigned a genetic identity. In our study these were ATC [33], *Cryptoperidiniopsis brodyi* [54], *Scrippsiella trochoidea* [51], *Oxyrrhis marina* [57], *Luciella masenensis* [53]. *Symbiodinium* ITS group names follow those of clade and subclades [39].

Strains that showed more than 2% divergence from any other strain in the database were labeled undetermined. Species names of single sequences were kept the same unless found to be less than 2% divergent from another strain in the database. Clonal and strain synonyms were aligned using MEGA version 4 [81] and pairwise distances calculated using uncorrected p-distance model.

## Supporting Information

Figure S1 **Neighbour-joining phylogenetic analysis of ITS DNA barcodes for all dinoflagellates from culture collections in this study and from GenBank as per** **Fig. 3** **, with tree labels.** Samples with DINO prefix belong to this study. Brackets represent species groups as identified using criteria described in methods and results. Abbreviations: GB: Sequence from Genbank.
(TIF)

Figure S2 **Neighbour-joining phylogenetic analysis of Gymnodiniales ITS DNA barcode groups from culture collections in this study and from GenBank as per** **Fig. 3** **, with tree labels.** Barcode groups are represented by vertical lines.
(TIF)

Figure S3 **Neighbour-joining phylogenetic analysis of** ***Heterocapsa*** **ITS DNA barcode groups from culture collections in this study and from GenBank as per** **Fig. 3** **, with tree labels.** Barcode groups are represented by vertical lines.
(TIF)

Figure S4 **Neighbour-joining phylogenetic analysis of** ***Symbiodinium*** **ITS DNA barcode groups from culture collections in this study and from GenBank as per** **Fig. 3** **, with tree labels.** Barcode groups are represented by vertical lines.
(TIF)

Figure S5 **Neighbour-joining phylogenetic analysis of** ***Alexandrium*** **ITS DNA barcode groups from culture collections in this study and from GenBank as per** **Fig. 3** **, with tree labels.** Barcode groups are represented by vertical lines.
(TIF)

Table S1 **List of all dinoflagellate strains used in this study with their new barcode identity.** Species identities are based on 2% species cut off value. Species highlighted in orange type show incongruities between strain names and barcode identities. Strain synonyms (SS) are given in column G. For *Symbiodinium*, culture collection names in parenthesis record the name given for their respective GenBank accession number. *Symbiodinium* chloroplast 23S genotypes (Cp) are shown in brackets.
(XLS)

Table S2 **A comparison of dinoflagellate PWD values from four barcode studies.** A: ITS, this study; B: ITS [20]; C: *COB* [12]; D: COI [11] and COI *Prorocentrum* (intraspecies only [58]). PWD were calculated by TVM_G model for *COB*, and uncorrected p-distances for all other barcodes. NA = data not available. Note the number of *Symbiodinium* taxa were recorded differently: this study and that of Stern *et al.* 2010 [11] identified a species by its smallest genotypic designation. Lin *et al.* 2009 [12] and Litaker *et al.* 2007 [20] used taxonomic species designations.
(XLS)
