# Supplementary figures and images for: Evaluating the Ribosomal Internal Transcribed Spacer (ITS) as a Candidate Dinoflagellate Barcode Marker
Source: PLoS One. 2012 Aug 16;7(8):e42780. doi: 10.1371/journal.pone.0042780 (PMC3420951; doi:10.1371/journal.pone.0042780)

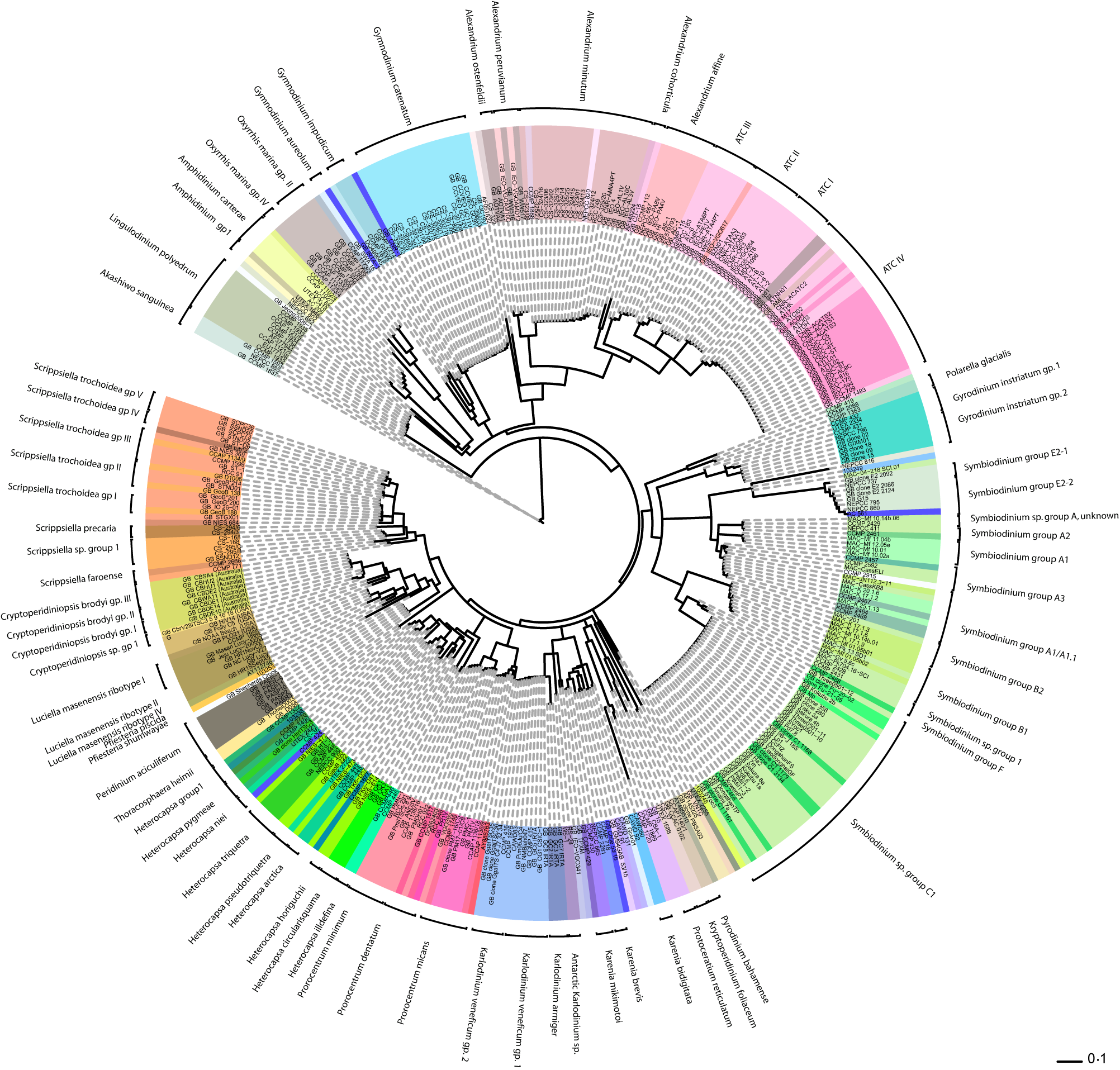

Supplement: Figure S1 — Neighbour-joining phylogenetic analysis of ITS DNA barcodes for all dinoflagellates from culture collections in this study and from GenBank as per Fig. 3 , with tree labels. Samples with DINO prefix belong to this study. Brackets represent species groups as identified using criteria described in methods and results. Abbreviations: GB: Sequence from Genbank. (TIF) [file pone.0042780.s001.tif]

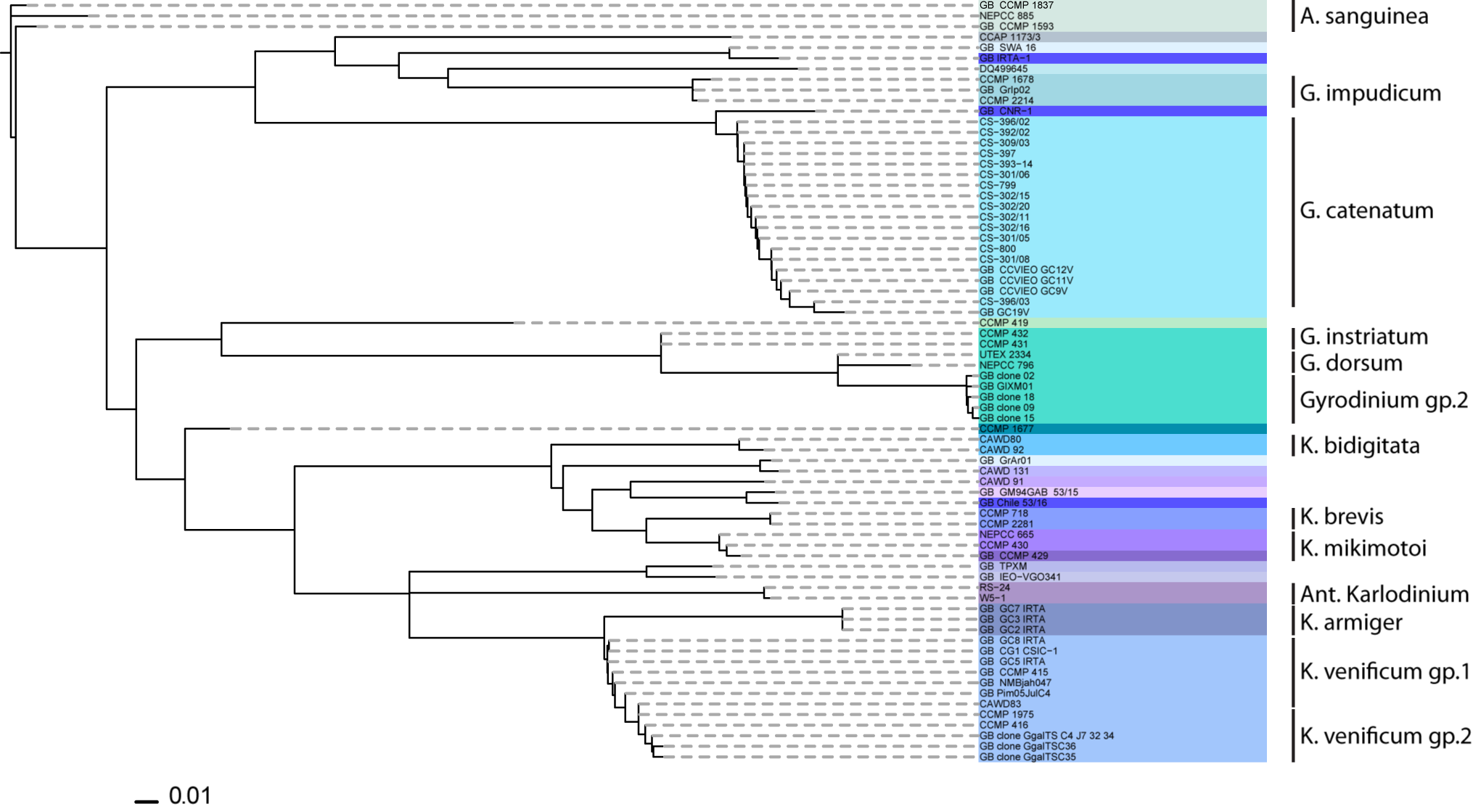

Supplement: Figure S2 — Neighbour-joining phylogenetic analysis of Gymnodiniales ITS DNA barcode groups from culture collections in this study and from GenBank as per Fig. 3 , with tree labels. Barcode groups are represented by vertical lines. (TIF) [file pone.0042780.s002.tif]

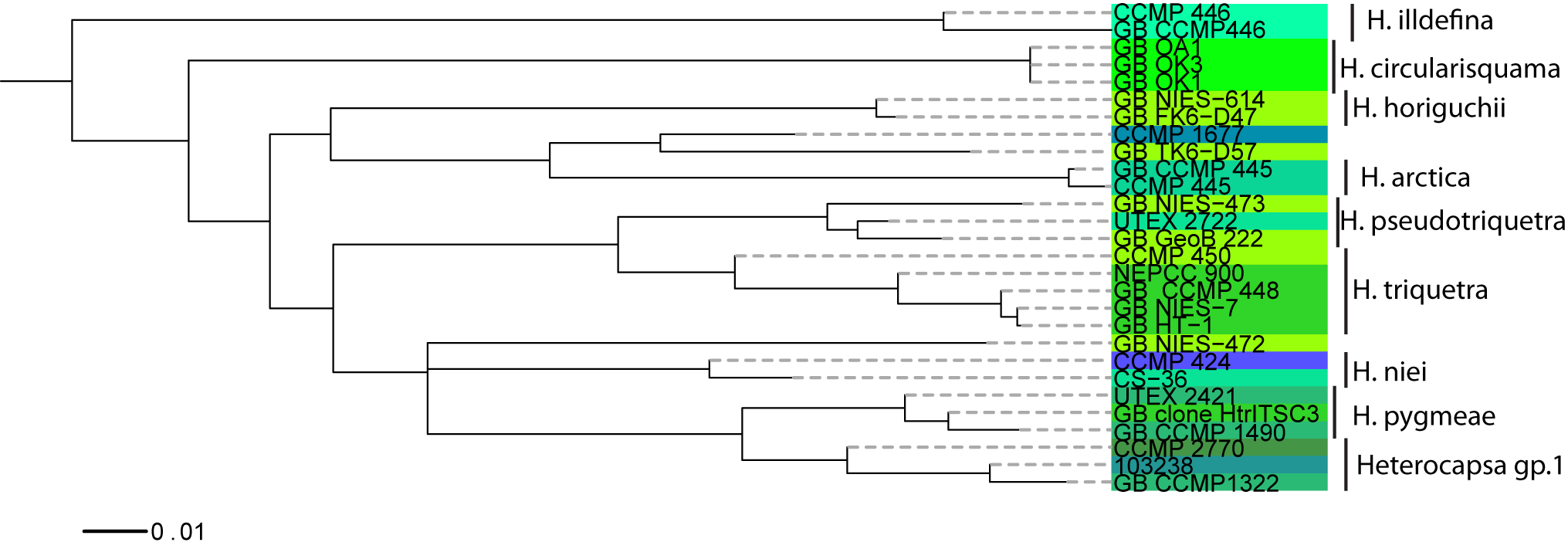

Supplement: Figure S3 — Neighbour-joining phylogenetic analysis of Heterocapsa ITS DNA barcode groups from culture collections in this study and from GenBank as per Fig. 3 , with tree labels. Barcode groups are represented by vertical lines. (TIF) [file pone.0042780.s003.tif]

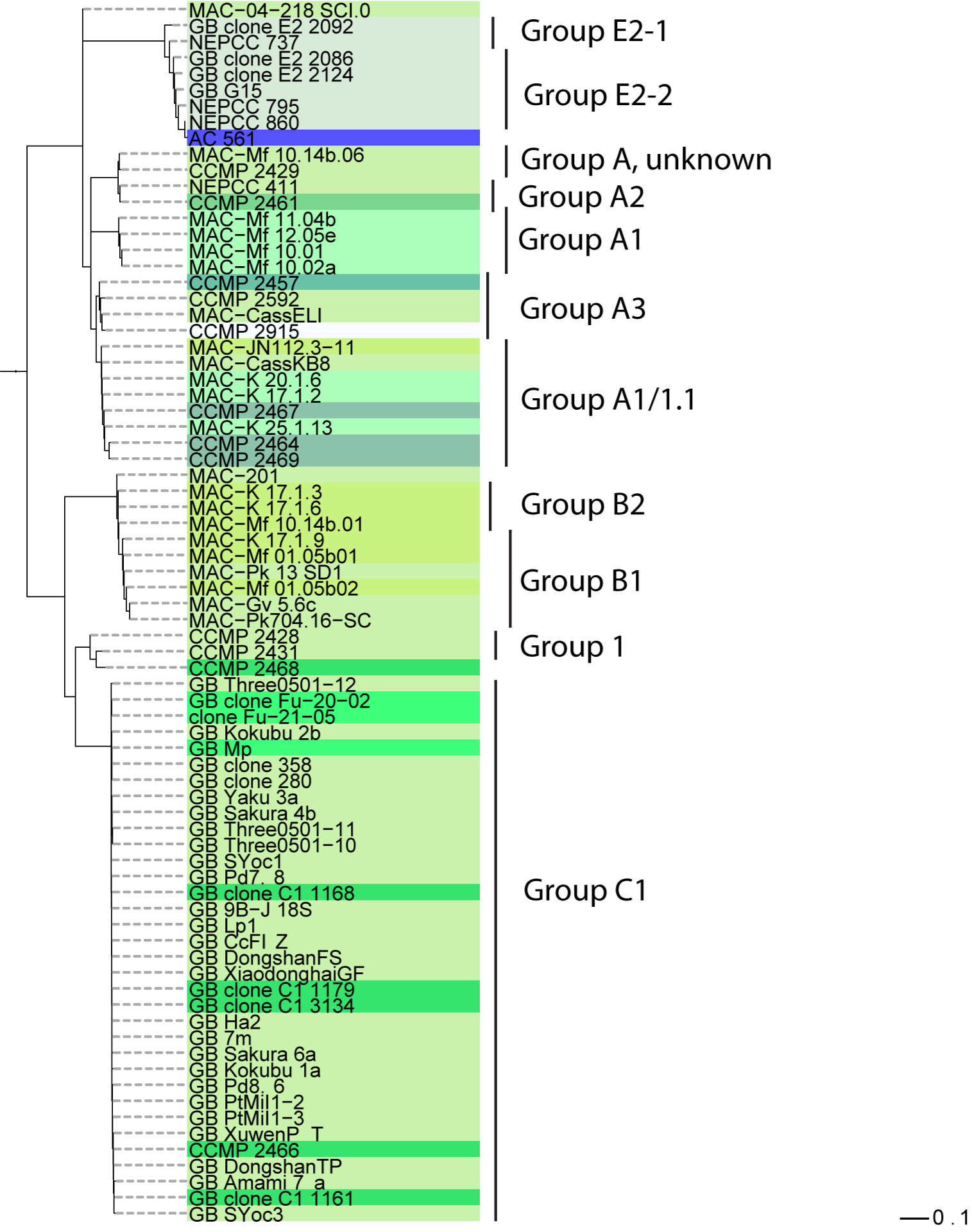

Supplement: Figure S4 — Neighbour-joining phylogenetic analysis of Symbiodinium ITS DNA barcode groups from culture collections in this study and from GenBank as per Fig. 3 , with tree labels. Barcode groups are represented by vertical lines. (TIF) [file pone.0042780.s004.tif]

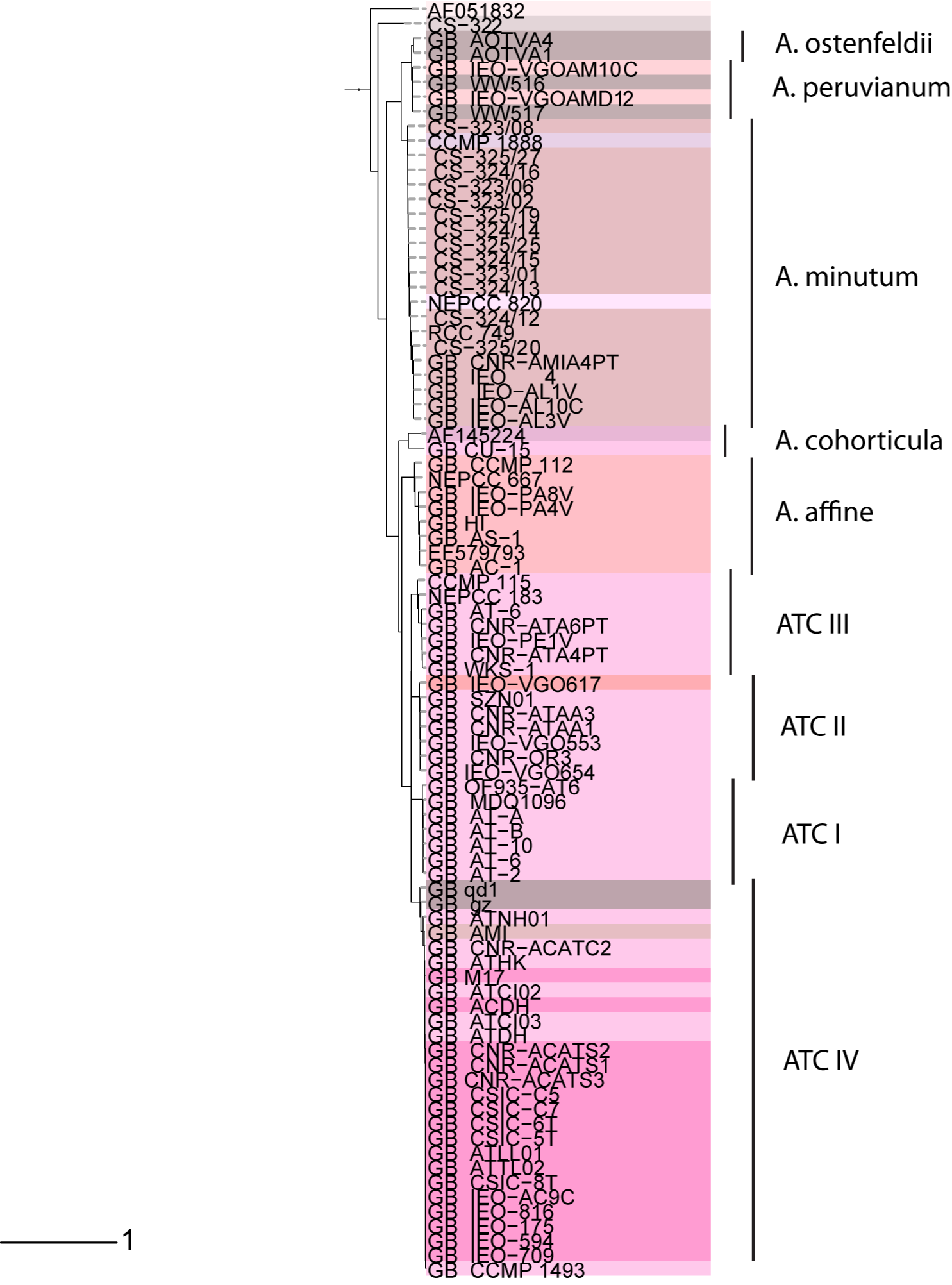

Supplement: Figure S5 — Neighbour-joining phylogenetic analysis of Alexandrium ITS DNA barcode groups from culture collections in this study and from GenBank as per Fig. 3 , with tree labels. Barcode groups are represented by vertical lines. (TIF) [file pone.0042780.s005.tif]
